# Comparative outcomes of focal HIFU versus active surveillance in low- and intermediate-risk localized prostate cancer: a 75-month retrospective cohort study

**DOI:** 10.3389/fruro.2026.1801127

**Published:** 2026-06-02

**Authors:** Mohammad Jaabou, Maurizio Buscarini, Osama Zaytoun, Zachary Dovey, Brooke Hildebrand, Modassar Awan, Maida Bada, Mohammad Al Ogaili, Ahmad Rabie

**Affiliations:** 1 Department of Urology, Hamah National Hospital, Hamah, Syria; 2 Mount Sinai Hospital, New York, NY, United States; 3 St. George’s University, St. George’s, Grenada; 4 Clínica Sagrada Familia, Barcelona, Spain; 5 Northwell Health, New York, NY, United States

**Keywords:** active surveillance, focal therapy, mp-MRI, prostate cancer, radical treatment

## Abstract

**Objective:**

Focal high-intensity focused ultrasound (HIFU) is a minimally invasive treatment for localized prostate cancer. Its comparative effectiveness against active surveillance (AS) remains underexplored in patients eligible for either strategy.

**Methods:**

This retrospective cohort study analyzed 635 men with localized prostate cancer treated between 2018 and 2023: 303 received focal HIFU and 332 underwent AS. Only low-risk and favorable intermediate-risk patients per NCCN guidelines were included (PSA <20 ng/mL, stage ≤T2b, ISUP ≤2, <50% positive cores). Multiparametric MRI and targeted biopsies confirmed eligibility. Outcomes included progression to clinically significant prostate cancer (csPC: ISUP ≥3 or ISUP 2 with PIRADS ≥4), radical treatment-free survival, PSA kinetics, and functional outcomes (IPSS, IIEF-5, continence).

**Results:**

Baseline characteristics were comparable. At 12 months, csPC-free survival was higher with HIFU in intermediate-risk patients (95.8% vs. 84.0%, p=0.0018) and low-risk patients (97.6% vs. 88.3%, p=0.0035). At a median follow-up of 75 months, HIFU maintained superior csPC-free rates in both risk groups. Radical treatment-free survival was similar in both low and intermediate risk group 76% vs 77.1% and 83.2% vs 80.3% respectively, time to radical treatment was significantly longer in the HIFU group. PSA reductions at 3 and 6 months were greater with HIFU. Functional outcomes, including erectile function, continence, and urinary symptoms, were comparable between groups.

**Conclusions:**

Focal HIFU offers better short-term oncological control and delays the need for radical treatment compared to AS, with similar preservation of urinary and sexual function. These results support its use in selected patients seeking a balance between cancer control and quality of life.

## Introduction

In 2020 prostate cancer remained the second most common cancer diagnosis, and the majority of cases, with modern diagnostic techniques, were localized at the time of presentation. Traditionally management options have included active surveillance for low-risk disease, and surgery or radiotherapy for intermediate and high-risk disease. Focal therapy (FT) has increasingly become a viable treatment for selected patients, although the patient population in whom its use is most effective remains controversial (1).

Moreover, surgery and radiotherapy are associated with urinary and erectile dysfunction significantly impacting patients’ quality of life such that younger patients with intermediate risk disease will often want to explore this as an option.

Despite increasing interest in FT, neither the AUA nor NCCN routinely recommend FT for any risk group because of lack of evidence. Nevertheless, there have been several published series as well as a significant increase in the number of patients treated with FT, with a reported 3230 men treated with FT from 1996 to 2015, increasing to 5827 from 2015 to 2020. Another retrospective study over 20 years found over three quarters of patients treated had GGG1 low risk disease, although a more recent 5 year study found patient characteristics had changed with over half having intermediate risk disease and a small proportion high risk disease (2).

This increasing interest has culminated in a consensus statement from the University of California collaborative, recommending all patients should be counselled that FT remains an investigative treatment, if possible, it should be done within a clinical trial, and patients should have localized GGG2 intermediate risk disease (or low volume GGG3 intermediate risk disease) and a life expectancy of over 10 years (stating the presence of GGG1 outside of the target lesion is not an exclusion factor) (3).

With regards to higher risk disease states, they recommend treatment with surgery or radiotherapy based on the lack of current evidence for FT use in this patient population. This consensus was recently emphasized at AUA 2025 and is part of the current AUA guidelines (4).

HIFU is the commonest energy source used for FT in the literature (2). It destroys malignant tissue by heat with intralesional temperatures of 65 degrees causing coagulative necrosis whilst also allowing real time evaluation of the necrotic border with contrast enhanced ultrasound (5).

The patient cohort presented in this multicenter study was prospectively collected and compares HIFU FT with deferred treatment/active surveillance in a group of patients with low and selected intermediate risk disease. 635 of patients were followed up for 75 months allowing a comparison of full treatment conversion rates, oncological and functional outcomes. Patients treated with HIFU underwent HIFU of the index lesion only. The presentation of this comparative restrospective study is timely as it provides an analysis of outcomes from a historical group of patients broadly representing different risk stratifications in direct comparison to a group of patients having deferred treatment and so contributes to our understanding of the optimal clinical setting for the use of FT and HIFU.

## Patients and methods

This retrospective cohort study was conducted to compare oncological and functional outcomes in men with localized prostate cancer who underwent focal therapy using high-intensity focused ultrasound (HIFU) versus those managed with active surveillance (AS). Patient data were collected between January 2018 and December 2023 from one center. The study was approved by the institutional ethics committee, and all procedures were performed in accordance with the Declaration of Helsinki and institutional guideline.

A total of 635 patients were included in the final analysis, comprising 303 patients managed with AS and 332 patients treated with HIFU.

Patients were classified into risk groups based on the highest of PSA, clinical stage, or ISUP grade, based on NCCN criteria. Low risk was defined as PSA < 10 ng/mL, ISUP 1, and T1–T2a stage; intermediate risk as PSA 10–20 ng/mL, ISUP 2, or T2b stage; High-risk patients were excluded. This classification was applied to both treatment groups — focal ablation therapy and active surveillance(AS) — to allow consistent comparison.

At the time of treatment decision-making, active surveillance (AS) was the institutional standard of care for patients with low-risk disease, in accordance with contemporary guidelines. Nevertheless, 37% of the patients in the active surveillance (AS) group and 41% of the patients in the HIFU group had low-risk disease, and treatment choice (HIFU versus AS) was based on shared decision-making between physician and patient, reflecting individual preferences and values. This approach was approved by the institutional ethics committee but was not a randomized allocation.

### Inclusion and exclusion criteria

Patients aged between 40 and 80 years were considered eligible if they had a PSA level <20 ng/mL and a clinical stage of cT1 or cT2b. Pathological eligibility required:

- ISUP grade 1 with a biopsy core length ≥5 mm, or
- ISUP grade 2 with less than 50% of biopsy cores positive for cancer.

All patients had histologically confirmed prostate-confined disease with no more than three distinct positive cores. Disease localization and staging were performed using multiparametric magnetic resonance imaging (mpMRI) of the prostate before the procedure. Imaging was performed on a 3.0 Tesla Siemens MRI scanner (Siemens Healthineers, Erlangen, Germany) using a pelvic phased-array surface coil without the use of an endorectal coil. multiparametric magnetic resonance imaging (mpMRI), including T2-weighted, diffusion-weighted, and dynamic contrast-enhanced sequences. Patients with biopsy-confirmed mpMRI lesions located <6 mm from the apex or <5 mm from the sagittal midline were excluded All images were interpreted by an experienced radiologist, and lesions were scored using the Prostate Imaging Reporting and Data System (PI-RADS) v2.1, with scores of 4 or 5 considered highly suspicious for clinically significant prostate cancer. Metastatic disease was ruled out using multi-slice computed tomography (CT) and bone scintigraphy. All pathological assessments were performed by dedicated genitourinary pathologists.

### Biopsy techniques

Initial biopsies included Targeted biopsy with At least two cores per lesion were taken using fusion-guided ultrasound or in-bore MRI guidance. And Systematic biopsy of A standard 12-core transrectal template.

Repeat biopsies were conducted during follow-up using the same approach as baseline. In the HIFU group, systematic sampling was also performed in untreated zones. Transperineal access was preferred for follow-up biopsies due to improved anterior zone sampling and reduced infection risk.

### HIFU procedure

#### Technique description

The procedure was performed under general anesthesia with the patient placed in the lateral decubitus position. Once the patient was prepped and draped in a sterile fashion, the Ablatherm^®^ HIFU system (EDAP TMS, Lyon, France) was used.

The transrectal ultrasound probe was prepared by covering it with a sterile condom and applying a generous amount of ultrasound gel to ensure proper acoustic coupling and to reduce friction. The lubricated probe was then gently inserted into the rectum and positioned to obtain optimal imaging of the prostate gland.

The acoustic power output at focus, set in the range of 30–50 W/cm², sufficient to raise the focal tissue temperature rapidly to 85–100 °C, resulting in immediate coagulative necrosis while avoiding thermal injury beyond the target.

The interval between pulses, was generally between 6–10 seconds, to allow surrounding tissues to cool before treating the next focal point, minimizing cumulative thermal spread.

Safety margins were maintained by keeping at least 3–4 mm distance from the urethra, rectal wall, and neurovascular bundles.

Care was taken to avoid thermal injury to surrounding tissues such as the rectal wall and neurovascular bundles.

Following completion of the ablation, the probe was removed, and the patient was monitored in the recovery area. Post-procedure care included routine observation, pain management, and instructions regarding urinary catheter care, along with follow-up plans for imaging, PSA monitoring, and clinical evaluation.

### Criteria for radical treatment

Patients were considered for radical treatment (surgery or radiotherapy) if they demonstrated disease progression during follow-up, defined as either ISUP grade ≥3 on repeat biopsy or ISUP grade 2 with PIRADS score ≥4 on mpMRI. Additional considerations included patient preference, age, comorbidities, PSA kinetics, and prostate volume. High-risk patients at diagnosis were excluded from this cohort. This approach ensured that decisions regarding radical treatment reflected both objective disease progression and individual clinical context.

### Follow-up and outcomes

Patients were monitored annually for up to 75 months or until they underwent definitive radical treatment due to disease progression. Follow-up was discontinued for 133 patients (20.9%) who proceeded to radical therapy, and their data were censored at the time of treatment initiation. The follow-up schedule—included PSA testing, multiparametric MRI, and targeted/systematic prostate biopsies at predefined intervals.

Oncological outcomes included:

- Progression to clinically significant prostate cancer (csPC), which defined as:

either ISUP grade ≥3, or ISUP grade 2 accompanied by a PI−RADS score ≥4 on multiparametric MRI. This definition was chosen to reflect both histologic and radiologic risk, allowing better identification of aggressive disease.

- Radical treatment-free survival.
- Time to radical treatment (e.g., radical prostatectomy or radiotherapy).
- Biochemical progression, including PSA variation with time.

Functional outcomes included Erectile function, assessed using the International Index Erectile Function Score (IIEF-5) at baseline and at the end of follow- up. Urinary symptoms, assessed using the International Prostate Symptom Score (IPSS). Incontinence status was assessed based on the number of pads used per day, with pad-free status defined as no pad usage and mild incontinence defined as the use of one safety pad per day. Follow-up assessments included serum PSA testing, mpMRI with PIRADS scoring, repeat prostate biopsies for ISUP grading, and administration of IIEF-5 and IPSS questionnaires.

### Statistical analysis

Continuous variables were summarized using mean ± standard deviation (SD) or median with interquartile range (IQR), depending on distribution. Comparisons between treatment groups were performed using the Student’s t-test for normally distributed continuous variables, Mann–Whitney U test for non-normally distributed or ordinal data, Wilcoxon signed-rank test for paired samples, and the chi-square test for categorical variables.

Kaplan–Meier curves were constructed to illustrate time-to-event outcomes in each treatment group. A two-sided p-value <0.05 was considered statistically significant. Statistical analyses were conducted using R software (version 4.4.1; R Foundation for Statistical Computing, Vienna, Austria).

## Results

Baseline characteristics, including age, BMI, PSA, prostate volume, and IIEF-5 scores, were comparable between AS and HIFU groups (**Table 1**).

**Table 1:** Subjects baseline characteristics.

Characteristic	Active surveillance (n = 332)	Focal ablation (n =303)	Overall (n=635)
Age, yr (mean ± SD)	60.8 ± 10.7	61.6 ± 10.3	61.2 ± 10.5
BMI (kg/m²)	29.3 ± 4.0	29.7 ± 4.2	29.5 ± 4.1
Prostate volume (ml) (mean ± SD)	69.0 ± 27.8	71.1 ± 27.7	70.0 ± 27.7
Serum PSA (ng/ml) (mean ± SD)	7.6 ± 3.0	7.5 ± 2.9	7.6 ± 2.9
IPSS PREOP (median +IQR)	16.0 (11.0)	14.0 (11.0)	15.0 (11.0)
IIEF-5 score PRE-treatment (median +IQR)	16.0 (9.0)	15.0 (10.0)	16.0 (9.5)
IIEF-5 score at end of follow-up (median +IQR)	15.0 (7.0)	14.0 (8.0)	15.0 (7.0)
ISUP Grade at diagnosis n (%)			
– Grade 1	157 (47.3%)	154 (50.8%)	311 (49.0%)
– Grade 2	173 (52.1%)	143 (47.2%)	316 (49.8%)
– Grade 3	2 (0.6%)	6 (2.0%)	8 (1.3%)
PIRADS score pretreatment n (%)			
– PIRADS 2	117 (35.2%)	92 (30.4%)	209 (32.9%)
– PIRADS 3	154 (46.4%)	142 (46.9%)	296 (46.6%)
– PIRADS 4	60 (18.1%)	69 (22.8%)	129 (20.3%)
– PIRADS 5	1 (0.3%)	—	1 (0.2%)
Clinical stage n (%)			
– cT1	223 (67.2%)	197 (65.0%)	420 (66.1%)
– cT2a	109 (32.8%)	106 (35.0%)	215 (33.9%)
Number of lesions n(%)			
1	103 (31%)	94 (31)	197[31)
2	146 (44%)	131 (43.2%)	277(43.6)
3	83 (25%)	78(25.7)	161(25.4)
NCCN Risk classification at diagnosis *			
Low risk	123 (37.1)	124 (40.9)	247 (38.9)
Intermediate risk	208 (62.65)	175 (58)	383(60.31)

AS, Active Surveillance; FA, Focal Ablation; BMI, Body Mass Index; PSA, Prostate-Specific Antigen; IPSS, International Prostate Symptom Score; IIEF, International Index of Erectile Function Score; ISUP Grade, International Society of Urological Pathology Grade; PIRADS, Prostate Imaging-Reporting and Data System; cT1, cT2a, Clinical Tumor Stage; SD, Standard Deviation; n, Number of patients,* 5 patients excluded due to high risk disease.

Functional outcomes: IPSS scores, urinary continence (>85% pad-free), and clinically significant erectile function decline (≥5-point decrease in IIEF-5) were similar between groups, indicating comparable preservation of urinary and sexual function.

**Figure 1:**
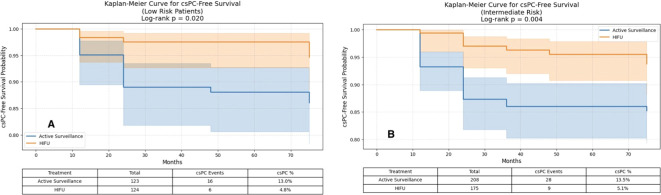
Clinically significant prostate cancer–free survival according to initial treatment and NCCN risk classification. **(A)** Low-risk patients. Kaplan–Meier curves comparing csPC-free survival between focal HIFU and active surveillance in patients classified as NCCN low risk. **(B)** Intermediate -risk patients. Kaplan–Meier curves comparing csPC-free survival between focal HIFU and active surveillance in patients classified as NCCN high risk.

Oncological outcomes: PSA remained stable in AS patients, while HIFU induced greater declines. Clinically significant prostate cancer (csPC)-free rates at 12 months and at final follow-up (median 75 months) were consistently higher in the HIFU group for both low- and intermediate-risk patients. Radical treatment-free survival was similar, though time to radical treatment was longer with HIFU. Kaplan-Meier analysis confirmed higher csPC-free survival in the HIFU group (**Figure 1**). Multivariable Cox regression confirmed that HIFU treatment and pre-treatment PSA were independent predictors of csPC, while age and ISUP grade at diagnosis were not (**Table 2**).

**Table 2:** Multivariable cox regression analysis of factors associated with csPC progression.

Variable	HR	95% CI	P value
Treatment (HIFU vs AS)	0.63	0.48-0.83	< 0.005
Pre treatment PIRADS score	2.63	2.14-3.23	<0.005
Age (years)	1	0.99-1.02	0.57
PSA PREOP (ng/mL)	1.23	1.18-1.29	0.003
ISUP grade at diagnosis	1.1	0.85-1.42	0.48

HR, hazard ratio; CI, confidence interval; csPC, clinically significant prostate cancer; AS, active surveillance; HIFU, high-intensity focused ultrasound.

## Discussion

Focal therapy using high-intensity focused ultrasound (HIFU) has evolved over the past two decades as a promising organ-preserving treatment for localized prostate cancer. Initially introduced as a whole-gland ablative approach, HIFU was associated with significant urinary and sexual morbidity, prompting a shift toward focal and hemi-gland techniques aimed at treating the index lesion while sparing surrounding tissue and minimizing side effects (6, 7). Despite these advances, several challenges remain, including operator dependence, the need for rigorous follow-up protocols—often involving repeat biopsies—and the lack of long-term oncologic data (8, 9).

Traditionally, HIFU has been offered to patients with low- to intermediate-risk localized disease who seek a less invasive alternative to radical treatments or are unsuitable for surgery (10, 11). Some centers have cautiously expanded its use to selected high-risk patients, although this remains controversial due to limited evidence and concerns about adequate oncologic control in more aggressive disease (12). Accurate patient selection through multiparametric MRI (mpMRI) combined with targeted biopsies is now widely recognized as essential for optimizing outcomes by localizing clinically significant lesions and excluding multifocal or high-volume disease (13, 14).

Our study is, to our knowledge, among the first to directly compare focal HIFU with active surveillance (AS) in low- and intermediate-risk patients. We observed comparable functional outcomes between groups, including similar rates of urinary continence and erectile function preservation, consistent with earlier reports that focal HIFU provides a favorable balance between cancer control and quality-of-life preservation (15). Preservation of sexual function remains a priority for many patients, and our findings align with prior studies demonstrating minimal impact on erectile function following focal therapy when nerve-sparing techniques are employed (8).

Both treatment groups showed reductions in severe lower urinary tract symptoms (LUTS), though a slightly higher proportion of HIFU patients experienced moderate symptoms post-treatment, likely reflecting thermal effects on periurethral tissues (13). These findings are consistent with previous reports indicating that focal HIFU’s urinary symptom profile is comparable to AS, reinforcing its suitability for patients prioritizing symptom control alongside cancer management (16).

Oncologically, focal HIFU demonstrated superior short-term clinically significant prostate cancer (csPC)-free survival compared to AS, consistent with earlier prospective studies reporting high early disease control after focal ablation (6, 17). A notable strength of our study is the use of a more nuanced definition of csPC, incorporating both histologic grade and imaging findings. While prior studies have often classified all ISUP grade 2 cancers as clinically significant, emerging evidence suggests that not all ISUP 2 disease warrants immediate intervention, particularly when MRI findings are low risk (18, 19). Therefore, we defined csPC as ISUP grade ≥3, or ISUP 2 with a PIRADS score ≥4, ensuring that only patients with higher-grade or MRI-visible disease were classified as having significant progression. This approach reflects evolving clinical practice and supports a more individualized assessment of risk, minimizing overtreatment of indolent disease (20, 21).

Our multivariable Cox regression analysis further confirmed that HIFU treatment independently reduced the risk of csPC progression (HR = 0.63, 95% CI 0.48–0.83, p < 0.005), whereas age, baseline ISUP grade, prostate volume, and number of lesions were not significant predictors. Pre-treatment PSA and PIRADS score remained strong independent risk factors for csPC, highlighting the importance of baseline tumor characteristics in guiding follow-up and management.

Interestingly, despite the clear reduction in csPC with HIFU, radical treatment rates were similar between HIFU and AS groups, and Cox regression showed no significant difference for time to radical treatment (HR = 0.77, 95% CI 0.55–1.09, p = 0.14). This apparent discrepancy likely reflects that decisions for radical therapy are influenced by multiple clinical and patient-driven factors—including patient preference, PSA kinetics, PIRADS progression, and physician recommendation—beyond histologic or imaging progression alone. Therefore, while HIFU effectively reduces early disease progression, it does not always translate into immediate reductions in radical treatment rates, consistent with the evolving understanding of focal therapy in personalized care.

In our study, baseline PIRADS score was strongly associated with the risk of progression to clinically significant prostate cancer. Each one-point increase in PIRADS significantly increased the hazard of progression (HR 2.63, 95% CI 2.14–3.23). These findings are consistent with previous studies demonstrating that higher PIRADS categories on multiparametric MRI are strongly predictive of clinically significant disease and progression during active surveillance (22, 23).

Although long-term differences narrowed, HIFU significantly prolonged the time to radical treatment compared with AS, offering patients longer treatment-free intervals and potentially preserving quality of life (15, 17). We also observed greater PSA reduction at early follow-up in the HIFU group compared to AS, with a median PSA decline of 11.8% at 3 months and 8.2% at 6 months in HIFU-treated patients, versus no change in the AS group. This early PSA response reflects the cytoreductive effect of focal ablation on the treated lesion and may provide additional reassurance to patients and clinicians during follow-up. Previous studies have similarly reported modest but measurable PSA reductions after focal therapy, although the role of PSA kinetics as a surrogate marker of efficacy remains under investigation (24).

Collectively, these findings support the emerging consensus that focal HIFU is a valuable option for patients with localized prostate cancer who prioritize quality of life and are willing to accept the possibility of retreatment (25). Looking forward, improvements in imaging, biomarkers, and artificial intelligence hold promise for enhancing patient selection and treatment precision in focal therapy (26, 27). Nonetheless, randomized controlled trials with longer follow-up are needed to establish HIFU’s definitive role in personalized prostate cancer management (28). The observed discrepancy between PI-RADS and ISUP grade at diagnosis in predicting csPC progression may be explained by the inherent limitations of biopsy-based grading. The PROMIS trial demonstrated that standard TRUS-guided biopsy may miss clinically significant prostate cancer, highlighting the risk of under-sampling and potential undergrading (6). As a result, ISUP classification at diagnosis may not accurately reflect the true biological aggressiveness of the tumor. In contrast, multiparametric MRI provides a more comprehensive assessment of tumor burden and spatial heterogeneity. In particular, higher PI-RADS scores have been associated with an increased risk of disease progression in patients undergoing active surveillance. This is supported by longitudinal data demonstrating that MRI-based assessment can better capture tumor dynamics over time and identify patients at higher risk of clinically significant progression (29).

### Limitations

This study has several limitations that should be acknowledged. First, the retrospective design may introduce selection bias, particularly in treatment allocation, as patients with more favorable clinical profiles may have been preferentially selected for HIFU or AS. Although stratification by ISUP grade at diagnosis was performed, unmeasured confounding variables may still influence outcomes. Second, while the follow-up period was adequate to assess mid-term functional and oncological outcomes, longer-term data are necessary to evaluate durability of cancer control, late recurrences, and the true impact on overall survival.

## Data Availability

The raw data supporting the conclusions of this article will be made available by the authors, without undue reservation.
